# Analysis of Mouse Brain Transcriptome After Experimental Duvenhage Virus Infection Shows Activation of Innate Immune Response and Pyroptotic Cell Death Pathway

**DOI:** 10.3389/fmicb.2018.00397

**Published:** 2018-03-20

**Authors:** Penelope Koraka, Byron E. E. Martina, Henk-Jan van den Ham, Fatiha Zaaraoui-Boutahar, Wilfred van IJcken, Jouke Roose, Geert van Amerongen, Arno Andeweg, Albertus D. M. E. Osterhaus

**Affiliations:** ^1^Department of Viroscience, Erasmus Medical Centre, Rotterdam, Netherlands; ^2^Viroclinics Biosciences B.V., Rotterdam, Netherlands; ^3^Artemis One Health Research Foundation, Delft, Netherlands; ^4^Erasmus Centre for Genomics, Erasmus Medical Centre, Rotterdam, Netherlands; ^5^Research Center for Emerging Infections and Zoonoses, University of Veterinary Medicine Hannover, Hannover, Germany

**Keywords:** rabies, Duvenhage virus, genomics, pyroptosis, innate immune response, caspase-1

## Abstract

Rabies is an important neglected disease, characterized by invariably fatal encephalitis. Several studies focus on understanding the pathogenic mechanisms of the prototype lyssavirus rabies virus (RABV) infection, and little is known about the pathogenesis of rabies caused by other lyssaviruses. We sought to characterize the host response to Duvenhage virus infection and compare it with responses observed during RABV infection by gene expression profiling of brains of mice with the respective infections. We found in both infections differentially expressed genes leading to increased expression of type I interferons (IFNs), chemokines, and proinflammatory cytokines. In addition several genes of the IFN signaling pathway are up-regulated, indicating a strong antiviral response and activation of the negative feedback mechanism to limit type I IFN responses. Furthermore we provide evidence that in the absence of significant neuronal apoptotic death, cell death of neurons is mediated via the pyroptotic pathway in both infections. Taken together, we have identified several genes and/or pathways for both infections that could be used to explore novel approaches for intervention strategies against rabies.

## Introduction

Rabies is an important neglected disease, caused by any of the viruses belonging to the *Lyssavirus* genus of the family *Rhabdoviridae*. There are at least 14 species within the genus. Rabies virus (RABV) is the prototype virus that has been found on all continents except Antarctica. The other members of the genus are geographically restricted and include: Lagos bat virus (LBV), Mokola virus (MOKV), Duvenhage virus (DUVV), Ikoma virus (IKOV), and Shimoni bat virus (SHIBV) all restricted to Africa; European bat lyssaviruses 1 and 2 (EBLV 1 and 2) and Bokeloh bat lyssavirus (BBLV), restricted to Europe; Australian bat lyssavirus (ABLV), restricted to Australia; Aravan virus (ARAV)

, Khujand virus (KHUV), Irkut virus (IRKV), and West Caucasian bat virus (WCBV) all restricted to Asia (Afonso et al., 2016). Lyssaviruses are mainly associated with bats. MOKV and IKOV are the only lyssaviruses that have not been associated with bats to date. Essentially all mammals can be infected with RABV, however, only a few species act as reservoir species and most commonly transmit the virus. Except bats, RABV is transmitted by carnivores mainly dogs, foxes, and raccoons. For the other lyssaviruses, bats are the main carriers while a spillover infection to other mammals seem to be sporadic.

Rabies is characterized by lethal encephalitis that accounts for at least 60,000 reported deaths annually, the majority of which occur in Africa (>40%) and Asia (>50%) (Fooks et al., 2014). No treatment for rabies exists and once symptoms occur the patient will die. Specific antibody preparations and vaccines against rabies for pre- or early post-exposure are available, however, they appear to be most effective against RABV infection. The post-exposure effectiveness of these biologicals against other lyssavirus infections either alone or in combination largely remains to be demonstrated. Similarly, several studies have been performed to elucidate mechanisms underlying the pathogenesis of RABV infection, but much less is known about the pathogenesis of clinical rabies caused by other lyssaviruses. DUVV is a lyssavirus that has been found in bats across southern Africa and has caused three documented lethal human infections (Markotter et al., 2013). The last human case of DUVV infection was documented in Kenya in 2007, in an area where bat rabies was not known to occur (van Thiel et al., 2008). The virus belongs to the same phylogenetic group as RABV, EBLV 1 and 2, and ABLV. DUVV causes similar clinical signs and lethality in experimentally infected mice as some lab-adapted RABV strains. This clinical picture contrasts the clinical signs and lethality caused by highly pathogenic wild type bat-associated RABV (Koraka et al., 2012). An additional contrast of DUVV compared to other lyssaviruses is that these species seem to have less heterogeneity on the nucleotide level compared to other lyssaviruses even when virus isolates were obtained decades apart (Markotter et al., 2008).

A large body of literature describes the (innate) immune response and the possible pathogenic mechanisms that lead to rabies due to RABV (reviewed extensively by Scott and Nel, 2016). A number of studies in mouse models have demonstrated the induction of different signaling pathways of innate response and programmed cell death after infection with RABV. Several other factors such as increased expression of nitric oxide, heat shock proteins, and glucocorticoids as well as mitochondrial dysfunction have been implicated in RABV pathogenesis (for references, see Scott and Nel, 2016). Contrary to that, there is still little knowledge on the pathogenesis that leads to the development of rabies upon DUVV infection either in the mouse model or after natural infection. Limited studies have been done to elucidate the role of the DUVV-P protein in the regulation of innate immune response (Wiltzer et al., 2014; Masatani et al., 2016). It remains unclear if programmed cell death plays any important role in the pathogenesis of DUVV as seen in RABV infections. To gain insight into the pathogenic mechanisms that govern DUVV infection we used gene expression profiling to study the host response of mice experimentally infected with DUVV in comparison with that against RABV infection.

## Materials and Methods

### Viruses and Animal Infections

All experiments involving live virus were performed in a biosafety level 3 containment lab according to Dutch legislation. The low passage DUVV-NL07 isolate used in this study as well as rabies virus strain Pasteur (RABV-PV) were propagated on human neuroblastoma cells SK-N-SH and titrated on BHK-21-C13 cells as previously described (Koraka et al., 2012). Virus stocks were inactivated with beta-propiolactone (BPL) overnight at 4°C and subsequently BPL was inactivated by incubating the viral suspensions at 37°C for 1 h. Complete inactivation of virus stocks was confirmed with complete absence of viral replication in BHK-21-C13 cells upon passaging and sub-passaging. Eight-week-old female BALB/c mice were infected with 10^4^ TCID_50_ intracranially. Groups of six mice were inoculated with the following preparations: DUVV-NL07, DUVV-NL07-BPL, RABV-PV, RABV-PV-BPL. One group of mice (mock) received culture supernatant from SK-N-SH cells prepared similarly to virus stocks. All animals were kept in isolator cages during the experiment, were fed *ad libitum* and had a 12-h day/night cycle. Animals were euthanized 5 days post-infection (d.p.i.) upon development of the first signs of rabies including ruffled fur and hunched back. Brains were collected and half the brain was stored in -80°C in RNA-later (Ambion) whereas the other half was stored in formalin at room temperature.

### Genomics

Brains stored in RNA-later were homogenized in Trizol Reagent (Invitrogen, Breda, Netherlands), and subsequently total RNA was isolated and purified using the RNeasy Mini Kit (Qiagen, Hilden, Germany): 250 μl of ethanol was added to the upper aqueous phase of the processed Trizol samples and directly transferred to the RNeasy spin columns for purification. RNA concentrations and OD 260/280 ratios were measured with the NanoDrop ND-1000 UV-VIS spectrophotometer (NanoDrop Technologies, Wilmington, United States). Assessment of RNA quality and purity was performed with the RNA 6000 Nano kit on the Agilent 2100 Bioanalyzer (Agilent Technologies, Palo Alto, CA, United States). RNA (200 ng) was labeled using the MessageAmp Premier RNA Amplification kit (Applied Biosystems) and hybridized to Affymetrix GeneChip^®^ Mouse 4302 Arrays (Affymetrix, Thermo Fisher Scientific, Bleiswijk, Netherlands), according to the manufacturer’s recommendations. Image analysis was performed using GeneChip Operating Software (Affymetrix). Microarray Suite version 5.0 software (Affymetrix) was used to generate .dat and .cel files for each experiment.

### Real-Time PCR

Presence of viral RNA in the brains of inoculated animals was confirmed with real-time RT-PCR using specific primers for RABV and DUVV species as previously described (Koraka et al., 2012). The expression level of selected genes of interest was confirmed with real-time qPCR. mRNA isolated as described above was reverse transcribed to cDNA using oligo dT primer and Superscript III reverse transcriptase (both from Invitrogen). cDNA was used as template for qRT-PCR using commercially available primer/probe sets (Applied Biosystems) and the 2 × PCR Master Mix (Roche). Copy numbers of each gene were calculated against the house-keeping gene β-actin following the formula 2^-ΔCt^ × 10^5^ where ΔCt = Ct_gene of interest_ - Ct_β-actin_. Gene expression levels were analyzed with a two-tailed, non-parametric Mann–Whitney test (Graph Pad Prism version 5). Values of *p* ≤ 0.05 were considered statistically significant.

### Immunohistochemistry

Brains were removed and fixed in 10% neutral-buffered formalin, embedded in paraffin, and sectioned at 4 μm. Immunohistochemical analysis for virus nucleoprotein was performed on brain sections using the streptavidin–biotin–peroxidase technique. Briefly, brain sections were deparaffinized in xylane, re-hydrated in descending dilutions of ethanol and incubated for 20 min in 0.3% H_2_O_2_ diluted in methanol to block endogenous peroxidase activity. Antigen retrieval was performed by incubation for 15 min at 121°C in citrate buffer (0.01 M, pH 6.0). Primary antibodies included rabbit anti-rabies NP (1:500 Rabies polyclonal DFA Reagent; CHEMICON), goat anti-caspase 1 p20 (1:25; Santa Cruz Biotechnology), rabbit anti-NeuN (1:500; Millipore), and rabbit anti-IBA1 (1:500; Wako Chemicals). Secondary antibodies with fluorescent labels included donkey-anti-rabbit IgG labeled with Alexa Fluor 488 (1:250; Invitrogen), donkey anti-goat IgG Alexa Fluor 488 (1:250; Invitrogen), and donkey anti-rabbit IgG Alexa Fluor 555 (1:250; Invitrogen). A streptavidin–biotin–peroxidase kit (UltraVision Large Volume Detection System Anti-polyvalent, HRP Lab Vision, United States) was used as secondary antibody (goat anti-polyvalent)/streptavidin enzyme complex and 3-amino-9-ethyl carbazole (AEC; Sigma) was used as a substrate. Sections were counterstained with Mayer’s hematoxylin and mounted wit Kaiser’s glycerin-gelatin. Vectashield Hard+Set mounting medium with DAPI (Vector Laboratories) was used as mounting medium in all cases in which the secondary antibody had a fluorescent label. Sections incubated without the primary antibody were considered as blank.

### Data Analysis

Probe-level data was normalized using quantile normalization and the transformed probe values were summarized into probe set values by the median polish method (Gautier et al., 2004) using brainarray probe sets and ensemble 86 genome annotation (Dai et al., 2005). Probe set wise comparisons between the experimental conditions were performed using limma (Phipson et al., 2016). Correction for multiple testing was achieved by applying a false discovery rate (FDR) of 0.05, calculated using the Benjamini–Hochberg procedure. Gene set analysis for cell death pathways was performed using the “fry” procedure (limma). The outcome has been visualized using the “barcodeplot” function, which plots the *t*-statistic for every gene in a particular contrasts and highlights genes that are a member of a specific gene set. Further downstream processing and interpretation of data including principal component was performed in R, a language for statistical computation.

Relevant canonical pathways, casual relationships, regulatory networks, and functions from the data set were generated with the Ingenuity Pathway Analysis (IPA) software^1^. This software uses the manually curated Ingenuity Knowledge Base database to define associations within a given dataset. We considered differences observed within the dataset to be relevant when more than twofold increase in expression values were measured for a given gene. The causal analysis tools of IPA were used to infer regulator networks upstream of gene-expression and predict downstream effects on biological functions and diseases (Kramer et al., 2014). For these analyses we used the modules Upstream Regulator Analysis (URA), Mechanistic Networks, Causal Network Analysis (CNA), and Downstream Effects Analysis within the IPA package.

## Results

### Infection of Mice

**FIGURE 1:**
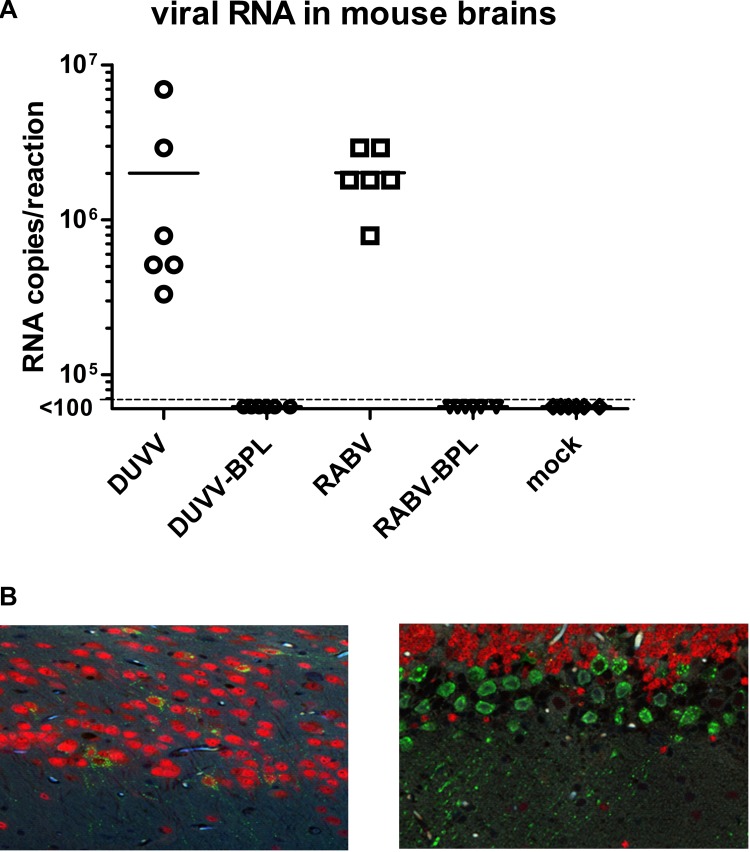
Infection of BALB/c mice with DUVV-NL07 and RABV-PV. **(A)** Viral RNA in the mouse brains at 5 d.p.i. Dotted line represents the limit of detection of the assay (<100 copies of RNA per reaction). **(B)** Extensive infection of pyramidal neurons of the hippocampus by DUVV-NL07 (left) and of the Purkinje cells of the cerebellum by RABV-PV (right). Tissues were stained with a polyclonal antibody that recognizes the N protein of RABV and observed at 20× magnification. Green, RABV antigen; red, NeuN staining for neurons.

All mice recovered within 2 h after intracranial viral inoculation under general anesthesia. At 5 d.p.i. mice that had received live DUVV-NL07 and RABV-PV viruses began to develop clinical signs including ruffled fur and hunched back but no signs of paralysis yet. At the onset of clinical signs the mice were sacrificed for gene expression analysis. Histological examination of the brains did not show any damage related to virus inoculation or replication. All mice infected with virus (DUVV-NL07 and RABV-PV) had detectable viral RNA (**Figure 1A**) and antigen (**Figure 1B**) in their brains at 5 d.p.i. confirming productive infection of the mice with either of the viruses. Some differences were observed in the RNA level of mice within the DUVV-NL07-infected group: there was at least a 10-fold difference in the low and high RNA levels measured in the DUVV-NL07 group. In agreement with previous publication (Koraka et al., 2012), differences in the distribution of viral antigen in the brains of DUVV-NL07-infected mice was observed compared to RABV-PV-infected mice. All animals inoculated with BPL-inactivated viruses as well as the mock inoculated animals failed to show clinical signs and tested negative for viral RNA (**Figure 1A**).

### Genomics Analysis

#### Global mRNA Profiling

**FIGURE 2:**
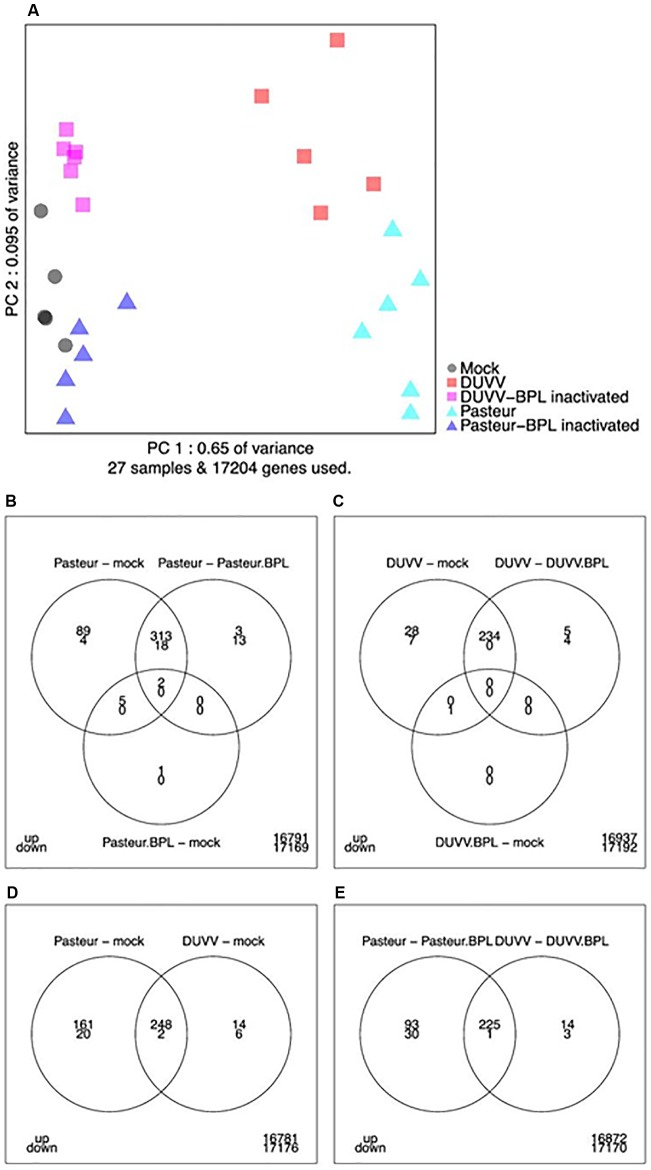
**(A)** Principal component analysis of transcriptome snapshots. The first and second components explain 65 and 9.5% of the variance. Different samples are indicated by colors. **(B–E)** Venn diagrams showing the number of up- and down-regulated genes applying a FDR threshold of 0.05 and fold change threshold of two (i.e., a 2-log-fold change of 1) for the indicated contrasts and overlaps.

Affymetrix mouse genome 430 2.0 arrays were used to compare gene expression profiles of mouse brains following infection with DUVV-NL07 or RABV-PV. Next, principal components analysis (PCA) was used to obtain a global view on lyssavirus infection induced transcriptome profile changes (**Figure 2A**). The first principal component accounts for 65% of the variance in the dataset whereas the second principal component accounts for 9.5% of the variance in the dataset. A distinct expression profile pattern of infected brains compared to mock-infected brains or brains that were inoculated with the respective BPL-inactivated viruses was observed. In addition, distinct RABV-PV- and DUVV-NL07-specific gene expression profiles could be identified both for virus-infected mice as well as for mice inoculated with BPL-inactivated virus preparations. The gene expression profiles of mock-infected mice bridged both BPL-inactivated virus groups (**Figure 2A**).

#### Differential Gene Expression Analysis

As shown in the Venn diagrams (**Figures 2B–E**), for most contrasts (pairwise comparisons of experimental conditions) the number of up-regulated genes was higher compared to the number of down-regulated genes. In addition, the results indicate that RABV-PV groups (both live virus and BPL-inactivated virus groups) have a more profound effect on gene expression in the brain compared to the respective DUVV-NL07 groups. In total, 262 genes were up-regulated and 8 down-regulated following DUVV-NL07 infection, whereas 409 genes were up-regulated and 22 were down-regulated following RABV-PV infection. The overlap between differentially expressed genes was pronounced between DUVV-NL07 and RABV-PV infections (**Figures 2B–E**). Approximately 95% of the genes differentially expressed after DUVV-NL07 infection were also differentially expressed after RABV-PV infection when comparing each to the mock-infected mice, indicating high similarities between the transcriptomic profiles of both infections. In contrast, only 61% of the genes that were differentially expressed following RABV-PV infection were also differentially expressed after DUVV-NL07 infection. This observation reflects the higher number of differentially expressed genes after RABV-PV, despite the presence of similar viral RNA loads in the brain of all mice (see below).

#### Gene Set Analysis

**FIGURE 3:**
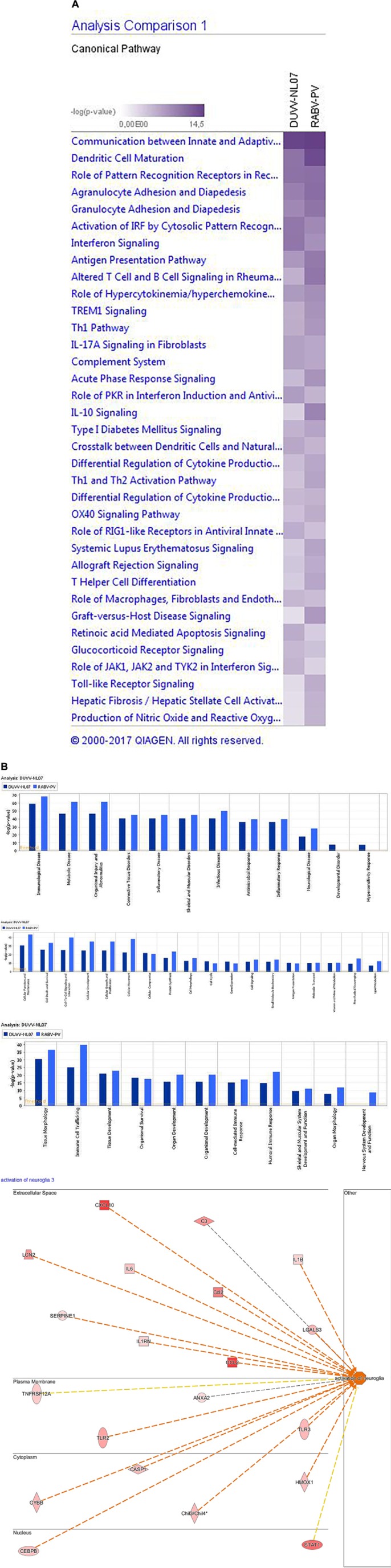
**(A)** Top canonical pathways identified by IPA. Picture depicts the comparison between enriched pathways after DUVV-NL07 and RABV-PV infection of mice based on their *p*-value. **(B)** Implication of differentially expressed genes in diseases and disorders (upper panel), molecular and cellular functions (middle panel), and physiological development and function (lower panel) identified by IPA. Activation of neuroglia cells is depicted in the insert of the lower panel as an example of nervous system development and function.

In addition to global analysis at the gene level, we also performed global gene set enrichment analysis using the canonical pathway tool from IPA. The *p*-value calculated for each pathway represents the likelihood for an observed association between a particular pathway (gene set) and an experimental condition. We identified the top canonical pathways that were enriched following DUVV-NL07 and RABV-PV infections. For both infections, these pathways were largely associated with immune responses and particularly innate responses (**Figure 3A**). Namely, communication between innate and adaptive immune cells, dendritic cell maturation, role of pattern recognition receptors (PRRs) in recognition of microorganisms, activation of IRF by cytosolic PRRs, interferon (IFN) signaling, antigen presentation pathway were among the canonical pathways identified for both DUVV-NL07 and RABV-PV with high *p*-values (Supplementary File 1). We used several tools of IPA to identify associations of our data set with different diseases and disorders, molecular and cellular functions, and biological and physiological functions. For both DUVV-NL07 and RABV-PV infections, differentially expressed genes were predominantly associated with innate immune response, cell death, and neurological disorders (**Figure 3B**).

When zooming into the individual genes within the data set, several PRRs were differentially expressed, including membrane PRRs such as TLR2 and TLR3, cytoplasmic PRRs such as MYD88, RIG-I, and MDA-5 and genes of the complement pathway such as C1qa, C1qb, C1qc, C3, and C4. Consequently, downstream IFN regulatory genes (including IRF1, IRF3, IRF7, and IRF8) were differentially expressed, collectively leading to increased expression of type I IFNs, chemokines (such as RANTES and CCL5), pro-inflammatory cytokines (such as IL-6 and IL-12). IFN signaling was also among the canonical pathways with low *p*-value with several genes within this pathway being up-regulated both in DUVV-NL07 and in RABV-PV-infected mice. Furthermore, we used the URA tool and the CNA tool of IPA to identify key molecules that regulate biological processes based on the expression data of our dataset. We identified several molecules that regulate activation of innate immune pathways (**Tables 1**, **2**). Master regulators of IFN signaling such as IFN receptors and STAT were predicted to be activated in our dataset, confirming that IFN signaling is activated during both infections of mice.

**Table 1:** Regulators of innate immunity and cell death identified by the URA tool of IPA.

Upstream regulator	Expression fold change	Molecule type	Predicted activation state	Activation *z*-score	*p*-value of overlap^∗^
IFNG	0.485	Cytokine	Activated	10.01	2.23E-89
Ifnar		Group	Activated	7.082	4.85E-74
STAT1	4.132	Transcription regulator	Activated	6.871	4.86E-74
IRF7	3.452	Transcription regulator	Activated	7.369	3.77E-70
IRF3	-0.028	Transcription regulator	Activated	6.454	3.99E-70
Interferon alpha		Group	Activated	7.302	9.84E-65
IFNB1	1.37	Cytokine	Activated	6.108	8.63E-60
IFNAR1	-0.02	Transmembrane receptor	Activated	4.98	3.28E-57
TLR4	0.204	Transmembrane receptor	Activated	6.496	2.46E-55
IRF1	2.769	Transcription regulator	Activated	5.459	8.06E-54
IFNA2		Cytokine	Activated	6.913	4.94E-52
TLR3	2.119	Transmembrane receptor	Activated	6.362	1.23E-50
TRIM24	-0.132	Transcription regulator	Inhibited	-6.422	2.24E-50
STAT3	1.244	Transcription regulator		1.609	2.64E-48
IL-1B	0.725	Cytokine	Activated	7.681	1.37E-44
TNF	0.376	Cytokine	Activated	8.728	6.11E-40
SOCS1	1.077	Other	Inhibited	-5.343	2.06E-38
TLR7	0.298	Transmembrane receptor	Activated	5.649	4.87E-38
MYD88		Other	Activated	6.16	8.67E-38
DOCK8	0.072	Other	Activated	5.568	9.26E-37
Ifn		Group	Activated	5.424	7.15E-36
IFN Beta		Group	Activated	5.507	1.84E-35
IFNL1		Cytokine	Activated	4.948	2.80E-35
IFN type 1		Group	Activated	4.271	3.72E-33
**DDX58**	**2.31**	**Enzyme**	**Activated**	**4.871**	**5.98E-33**
FADD	-0.124	Other		1.911	3.09E-25
STAT2	1.791	Transcription regulator	Activated	2.785	3.39E-19
Tlr		Group	Activated	4.219	3.70E-19


*Shown for DUVV-NL07 largely overlapping with regulators in RABV-PV. DDX58 is highlighted in bold as relevant mediator of cell death. ^∗^*p*-value of overlap measures overlap between dataset genes and the genes that are regulated by the depicted regulators.*

**Table 2:** Regulators of innate immunity and cell death identified by the CNA tool of IPA.

Master regulator	Expression log ratio	Molecule type	Depth	Predicted activation state	Activation *z*-score	*p*-value of overlap^∗^
IRF3	-0.028	Transcription regulator	2	Activated	10.076	9.07E-103
IFNG	0.485	Cytokine	1	Activated	9.898	1.34E-95
Ifnar		Group	2	Activated	9.857	1.02E-91
DHX58	0.809	Enzyme	2	Inhibited	-9.966	3.44E-90
IRF3-IRF7		Complex	2	Activated	7.506	8.95E-88
Tlr11		Transmembrane receptor	2	Activated	10.488	1.44E-82
TLR10		Transmembrane receptor	2	Activated	10.149	6.29E-81
Tlr13	0.165	Other	2	Activated	10.149	6.29E-81
Tlr12	-0.025	Other	2	Activated	10.149	6.29E-81
Interferon beta-1a		Biologic drug	2	Activated	7.275	5.09E-77
IRF3	-0.028	Transcription regulator	1	Activated	6.455	3.10E-74
Ifnar		Group	1	Activated	7.211	4.85E-74
PARP9	3.241	Enzyme	2	Activated	6.765	5.71E-74
IFNL4		Cytokine	2	Activated	6.862	5.08E-72
Stat1 dimer		Complex	2	Activated	7.608	5.82E-71
Stat1–Stat2		Complex	2	Activated	6.791	8.92E-71
IRF7	3.452	Transcription regulator	1	Activated	7.483	1.71E-69
STAT1	4.132	Transcription regulator	1	Activated	6.719	2.36E-69
Interferon alpha		Group	1	Activated	7.344	1.07E-66
TLR2/3/4/9		Group	2	Activated	8.393	1.75E-63
IFNA2		Cytokine	1	Activated	7.071	1.19E-54
TRIM24	-0.132	Transcription regulator	1	Inhibited	-6.481	9.58E-54
IFN alpha receptor		Complex	2	Activated	6.332	3.02E-50
MAP3K21	-0.144	Kinase	2	Activated	6.647	1.24E-47
IL-1B	0.725	Cytokine	1	Activated	7.951	7.04E-37
**DDX58**	**2.310**	**Enzyme**	**1**	**Activated**	**5.000**	**2.54E-33**
NLRC5	2.813	Transcription regulator	1		1.414	5.71E-12


*DDX58 is highlighted in bold as relevant mediator of cell death. ^∗^*p*-value of overlap measures overlap between dataset genes and the genes that are regulated by the depicted master regulators.*

From the differentially expressed genes detected, approximately 200 genes were differentially expressed uniquely in RABV-PV- and not in DUVV-NL-infected mice. These genes were mainly involved in inflammatory response, antigen presentation, and signaling for adaptive immune responses.

#### Induction of Anti-viral Responses

In DUVV-NL07 compared to RABV-PV-infected mice several genes of the IFN-signaling pathway proved to be more up-regulated, indicating a more prominent innate antiviral response. Several pathogen recognition receptors such as RIG-I, MDA, and TLR3 were up-regulated resulting in the induction of downstream genes such as IRF7 and TBK1 which lead to the production of type I IFNs. We did not observe significant up-regulation of the IFN receptors in our data set, however, downstream genes were up-regulated in DUVV-NL07-infected mouse brains. These genes included JAK1, STAT1, STAT2, and STAT3 leading to antiviral response (with the induction of Mx1 and OAS molecules) and inflammatory response (with the induction of CXCL9 and IRF1). Suppressor of cytokine signaling-3 (SOCS3) and USP18 were also found up-regulated, indicating that the negative feedback mechanism to limit type I IFN responses was activated in the brains of DUVV-NL07-infected mice. Interestingly, Eukaryotic Translation Initiation Factor 2-Alpha Kinase 2 (eIF2AK2, also known as PKR), OAS1, and IFN stimulated genes 20 and 56 (ISG20 and ISG56) involved in ISG-mediated suppression of protein synthesis in eukaryotic cells were also up-regulated in DUVV-NL07-infected mice. Collectively, these data suggest that the type I IFN signaling pathway is not impaired during DUVV-NL07 infection.

#### Activation of Cell Death Pathways

**FIGURE 4:**
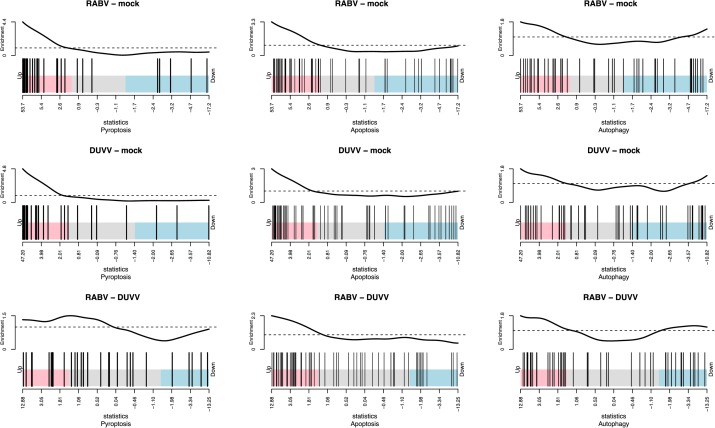
Enrichment index for pyroptosis, apoptosis, and autophagy after DUVV-NL07 and RABV-PV infection of BALB/c mice.

The pathogenesis of RABV infections is hypothesized to be dominated by neuronal dysfunction rather than significant neuronal death. Neither apoptosis, nor necrosis have been shown to be relevant in the pathogenesis of lyssavirus infections in mouse models (Jackson et al., 2008; Suja et al., 2009). The transcriptomic signatures in DUVV-NL07- and RABV-PV-infected mice showed no evidence of CASP-3- or CASP-9-induced apoptosis. Interestingly, a network of genes involved in the pyroptotic cell death pathway was up-regulated in the brains of infected mice (**Table 3**). Pyroptosis is induced upon activation of different PRRs, namely NOD-like receptors (NLRs), RIG-I, AIM2, or MDA-5. Activation of any of these receptors will lead to formation of the pyroptosome, activation of CASP-1 and production of IL-1β and IL-18. Both DUVV-NL07- and RABV-PV-infected mice showed increased expression mRNA levels of molecules involved in the formation of inflammasomes such as PYCARD, RIG-I, and MDA5. In addition, Gasdermin D, CASP-1 and CASP-4 (key molecules in the activation of pyroptotic pathways) mRNA was also elevated. To generate hypotheses as to how pyroptosis is induced in mouse brains upon DUVV-NL07 infection we used the URA tool of IPA (to predict the cascade of upstream networks that would explain our dataset observations) and the CNA tool of IPA (to generate a more complete picture of the possible upstream networks that cause our dataset observations). Both these tools identified RIG-I-mediated pyroptosis as being the most likely mechanism of cell death in the brains of DUVV-NL07- and RABV-PV-infected mice (**Tables 1**–**3**). To further support our observations, we compared the enrichment index of three major cell death pathways—pyroptosis, apoptosis, and autophagy—in our data set for the different viruses. As shown in **Figure 4**, both DUVV-NL07 and RABV-PV had higher enrichment indices for pyroptosis (4.8 and 4.4, respectively) compared to apoptosis (3 and 3.3, respectively) or autophagy (1.8 for both viruses).

**Table 3:** Expression levels of genes involved in pyroptotic cell death after DUVV-NL07 and RABV-PV infection of mice.

Gene	Fold change DUVV-NL07/RABV-PV
CASP1	1.5/2.1
CASP7	0.8/0.8
CASP4	1.7/2.4
PYCARD (ASC)	1.6/1.8
AIM2	0/0
NOD1	1.1/1.3
**RIG-1 (DDX58)**	**2.3/2.4**
IL-1B	0.8/1.3
IL-18	0.6/0.5
NLRC5	2.8/3.1
NLRC3	0/0
NLRC4 (IPAF)	0/0
**DDX60**	**2.3/2.7**
NLRP3	0/0
MDA5 (IFIH1)	4.3/4.3
GBP2	4.5/4.8
CASP11	0/0
Gasdermin D	1.0/1.2


*DDX58 and DDX60 are highlighted in bold as relevant mediator of cell death.*

**Table 4:** Mean transcript numbers relative to β-actin per nanogram of RNA of key pyroptotic genes measured by real-time qRT-PCR in the brains of the different mouse groups.

Mouse group	CASP-1	IL-1β	IL-18
DUVV-NL07 i.c. (BALB/c)	6.42	12.8	28.8
DUVV-NL07 BPL i.c. (BALB/c)	0.8	1.24	12
RABV-PV i.c. (BALB/c)	6.65	20.1	19.3
RABV-PV BPL i.c. (BALB/c)	1.4	1.9	16.6
DUVV-NL07 i.c. (Swiss)	7.41	6.76	10.2
CVS-11 i.c. (Swiss)	7.24	2.04	5.9
Controls i.c	2.04	0.76	28.8
DUVV-NL07 i.m (BALB/c)	15.5	28.2	170
RABV-PV i.m. (BALB/c)	17.8	24.5	158
SHBRV-18 i.m. (BALB/c)	61.7	33.1	1047
SHBRV-18 i.m. (C57BL/6)	170	38.0	302
Controls i.m.	7.08	7.24	81


**FIGURE 5:**
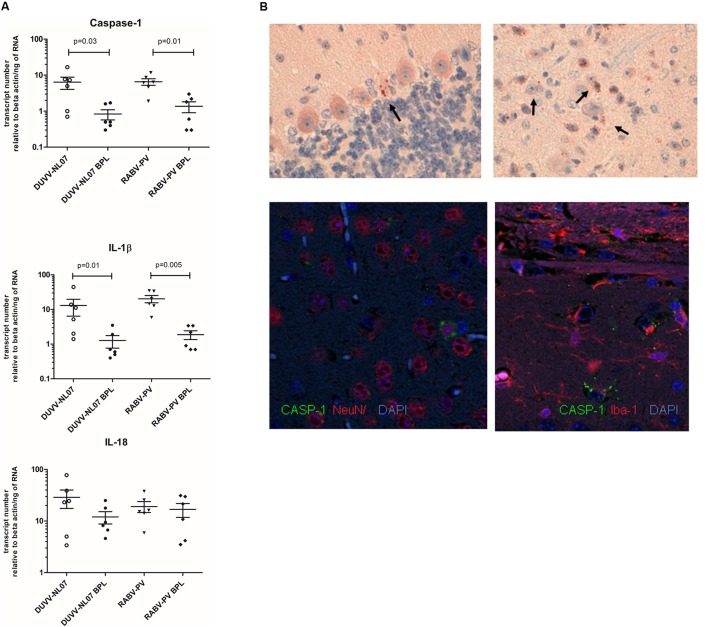
Confirmation of pyroptotic pathway in mice infected with DUVV-NL07 and RABV-PV. **(A)** Genomics data were confirmed by qRT-PCR for key pyroptotic molecules. Means and standard error of the mean are depicted in the plots. Actual mean numbers are given in **Table 4** for each gene. When differences are significant the *p*-value is given (Mann–Whitney non-parametric test). **(B)** Detection of p20 subunit of CASP-1 in mouse brains. CASP-1 was found in the mouse cerebellum (upper left panel) and cortex (upper right panel) of mice infected with DUVV-NL07. CASP-1 signal co-localized with NeuN (lower left panel) but not with IBA1 (lower right panel) indicating that neurons are the main producers of CASP-1.

**FIGURE 6:**
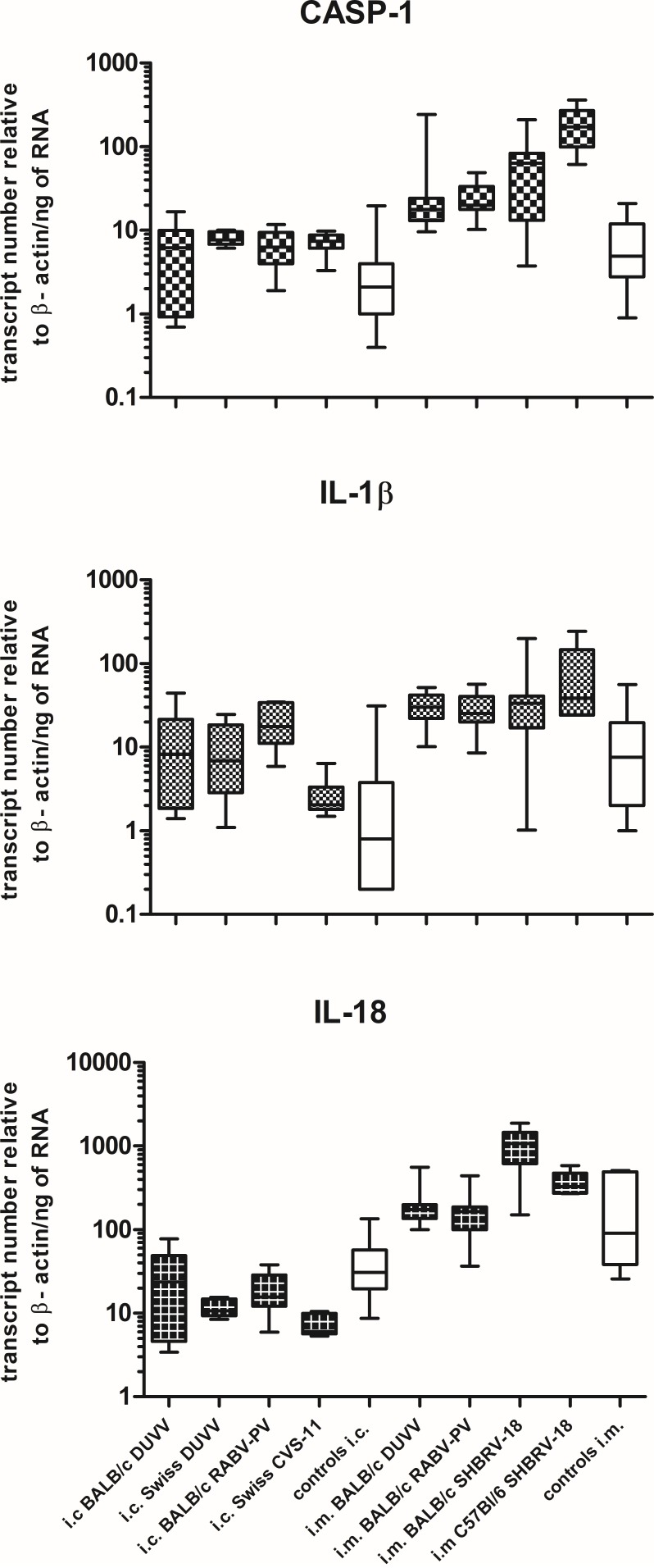
Pyroptosis is activated in different mouse strains after infection with different lyssaviruses. Shaded boxes represent virus-infected mice of the respective mouse strains. Open boxes represent pools of BPL and/or mock-inoculated mice of the respective mouse strains. Actual mean numbers are given in **Table 4** for each gene.

*Averages are taken from **Figures 5A** and **6A**.*

Using qRT-PCR, we confirmed that CASP-1, IL-1β, and IL-18, the three key molecules of the pyroptotic pathway, were indeed expressed in the brain of infected mice whereas BPL-inactivated virus did not result in the expression of these genes (**Figure 5A**). mRNA levels of CASP-1 and IL-1β were significantly higher in the groups that received infectious virus compared to the BPL-inoculated mice (for CASP-1: *p* = 0.034 and *p* = 0.012 for DUVV-NL07 and RABV-PV, respectively and for IL-1β: *p* = 0.015 and *p* = 0.0049 for DUVV-NL07 and RABV-PV, respectively; Mann–Whitney non-parametric test). There was no difference in the mRNA levels of CASP-1, IL-1β and IL-18 between mice infected with DUVV-NL07 or RABV-PV. In order to confirm that mRNA expression corresponded to the activated protein we performed immunohistochemistry to detect the p20 subunit of CASP-1. As shown in **Figure 5B**, CASP-1 was found in the brains of DUVV-NL07-infected animals and was particularly expressed in neurons (**Figure 5B**, lower panel) rather than in microglia or astrocytes (data not shown).

Consistent with transcriptome data, qRT-PCR for CASP-3 and CASP-9 showed no difference between these two key apoptotic molecules between infectious virus (DUVV-NL07 or RABV-PV) and their respective BPL controls (**Supplementary Figure S1**). qRT-PCR also confirmed the increased expression of TNF-α mRNA between infectious viruses and BPL controls (*p* = 0.004 for DUVV-NL07 vs BPL and RABV-PV vs BPL). TNF-α is a gene that is involved in apoptosis but is also expressed under several other conditions, especially in inflammatory and anti-viral host response.

#### Activation of the Pyroptosis Pathway in Lyssavirus Infection

This is the first evidence that pyroptotic cell death plays a role in rabies pathogenesis. To test for the presence of key pyroptotic molecules, we used retrospectively collected brain samples obtained from BALB/c mice that had been infected intramuscularly and succumbed to rabies encephalitis. As shown in **Figure 6**, CASP-1, IL-1β, and IL-18 were also expressed in the brain of mice with advanced rabies encephalitis (infected intramuscularly), at higher levels compared to the values that we obtained from the mice that had been infected intracranially and euthanized before advanced signs of encephalitis appeared. To obtain additional evidence for the involvement of this pathway in rabies encephalitis, we tested historical samples from different mouse strains infected with different rabies viruses. In detail, we tested samples from BALB/c, C57BL/6, and Swiss albino mice infected with either DUVV-NL07, RABV-PV, silver-haired bat rabies virus (SHBRV-18), or challenge virus standard (CVS-11). CASP-1 and IL-1β expression was increased in all samples from mice inoculated intracranially. In addition, increased expression of the three key pyroptotic molecules was observed after intramuscular inoculations (**Figure 6**). Consistently, mice with advanced encephalitis (i.e., after i.m. inoculations) had higher levels of transcripts compared to mice infected intracranially. These results were independent of the mouse or virus strain used. Taken together, our data suggest that pyroptosis is a pathogenic mechanism of lyssavirus-induced encephalitis (**Figure 6**).

## Discussion

The pathogenic mechanisms that eventually lead to lethal rabies encephalitis are poorly understood. Histopathological analyses of the brains of human and experimentally infected animals show minor to mild pathology. Furthermore most of the studies to elucidate rabies pathogenesis have been largely focused on classical RABV infection, whereas pathogenic mechanisms of rabies encephalitis caused by other lyssavirus infections and especially by DUVV have received little attention.

In the present study, we have investigated mRNA expression profiles of brains of BALB/c mice experimentally infected with DUVV-NL07 or with lab adapted RABV-PV. Since the kinetics of neuro-invasion by these two viruses upon peripheral inoculation differ, we chose the i.c. route to synchronize the infections. We have observed that the spread of viral RNA in the brains of DUVV-NL07-infected mice was more than 10-fold greater than the spread of viral RNA in RABV-PV-infected mice (**Figure 1A**). In the PCA, we did not notice any influence of this difference in the transcriptome profile of the different mice (**Figure 2**). To our knowledge, this is the first study to describe mRNA profiles in mice after infection with a lyssavirus other than RABV. Overall, the transcriptomic profiles of the two lyssaviruses did not differ significantly, although we observed a higher amount of differentially expressed genes after RABV-PV infection compared to DUVV-NL07 infection. We cannot fully exclude that the kinetics of the two virus infections upon i.c. infection differ, as was the case upon peripheral inoculation (Koraka et al., 2012). Another possible explanation to our observations is that the differences in numbers of differentially expressed genes reflects differences in pathogenicity of the two viruses or could be viewed as markers of lab adaptation (since RABV-PV is a lab-adopted strain and DUVV-NL07 a wild type virus). We also cannot exclude the possibility that these differences are due to different antigen distribution in the brains of infected mice (**Figure 1B**). Nevertheless, differentially expressed genes, enriched pathways and mechanistic networks largely overlapped between DUVV-NL07- and RABV-PV-infected brains. We previously showed that these two infections show many phenotypic similarities (Koraka et al., 2012). Here we provide evidence that also the mRNA profiles in the brains of infected mice are similar, suggesting a similar pathogenesis of both virus infections.

One of the first lines of host defense to invading viruses are IFNs, secreted by the majority of cells in response to viral infection. Productive infection will lead to viral replication and production of intermediate dsRNA, which is recognized by PAMPs with subsequent production of type I IFNs (Akira et al., 2006). While in the periphery plasmacytoid dendritic cells are the main producers of type I IFNs, in the CNS it has been demonstrated that neurons can also produce type I IFNs upon viral infection (Delhaye et al., 2006). While there is consensus that in mice experimentally infected with attenuated, lab adapted RABV strains IFN-signaling is activated, there is conflicting evidence whether this also is the case upon wild type RABV infection of mice (Wang et al., 2005; Zhao et al., 2011). A recent study demonstrated that the P protein of DUVV is not as strong antagonist of IFN signaling via STAT as is the P protein of RABV (Wiltzer et al., 2014). Our data show up-regulation of several IFN-associated genes, suggesting that type I IFN pathway is activated during DUVV-NL07 infection resulting in an antiviral response (as evident by the increased expression of Mx1 and OAS) (Ivashkiv and Donlin, 2014). Innate responses are not restricted to production of type I IFNs. Viral envelope proteins may activate certain TLRs to produce inflammatory responses. In addition to type I IFNs, DUVV-NL07 infections led to increase in expression of inflammatory markers such as IRF1 and CXCL9 (Akira et al., 2006). Despite the strong anti-viral response measured at the mRNA level, mice still succumb to rabies. It cannot be excluded that despite the increased mRNA levels of several genes of the type I IFN pathway, active protein is still not produced in a timely fashion. Furthermore, it has been shown that exogenous IFN given in RABV-infected cell lines does not prevent infection but rather enhances viral replication (Chopy et al., 2011). Other viruses, like Chikungunya virus are sensitive to the effect of IFN but only early after infection (Fros et al., 2010). On the other hand, many pathogens employ negative feedback mechanisms to counteract the effects of IFN responses. For instance, IFN inhibitory proteins such as SOCS and USP18, or activated MAPK pathways act as negative regulators for pathogens to escape antimicrobial responses (Ivashkiv and Donlin, 2014). Indications for such negative feedback mechanisms were also found activated in DUVV-NL07- and RABV-PV-infected brains, indicating that lyssaviruses have evolved mechanisms to evade host innate immune responses.

We have previously shown that DUVV-NL07 despite being a wild type virus isolated from a human fatal case, exhibits a virulence profile similar to attenuated viruses when studied in experimental animals (Koraka et al., 2012). Here, we confirm that also at the genomic level, DUVV-NL07-infected mice show an attenuated mRNA signature of the innate immune response. Only three human cases of DUVV infection have been described and therefore, it is virtually impossible to compare our data of experimentally infected mice with those of DUVV infected humans.

The major finding of our studies may well be that infection of mice with lyssaviruses results in a gene expression profile that involves pyroptosis. Pyroptosis, is a well-defined programmed cell death pathway that is highly dependent on the activation of Gasdermin D and subsequently, activation of the inflammatory caspases 1, 4, and 11 (reviewed in Aglietti and Dueber, 2017). Pyroptosis may be triggered by both canonical and non-canonical activation of the inflammasome (Kayagaki et al., 2011). Non-canonical activation of the inflammasome includes activation of CASP-11 and NLRP3. In the absence of activated CASP-11, formation of inflammasome may be triggered by activation of different molecules such as AIM2, RIG-I, and MDA5 in addition to NLRP3. Our data provide evidence that during lyssavirus infection, formation of the inflammasome is RIG-I-rather than NLRP3-mediated, without significant up-regulation of CASP-11. This suggests that activation of the inflammasome occurs via the canonical pathway. This observation is in agreement with previous reports describing RIG-I-mediated inflammatory response in human primary astrocytes after infection with vesicular stomatitis virus, another member of the *Rhabdoviridae* family (Furr et al., 2010; Poeck et al., 2010).

Recent studies have demonstrated the cross talk between different programmed cell death pathways. For example, CASP-8—a main effector molecule of apoptosis—can also play a key role in suppressing necroptosis (Oberst et al., 2011). Also apoptosis-associated speck like protein containing CARD (ASC), a key protein complex in the formation of the pyroptosome can induce apoptosis (Masumoto et al., 2003; Chung et al., 2016). In addition, IFN signaling may regulate pyroptotic cell death. For instance, repression of the inflammasome can be STAT1 or STAT3 mediated (Tschopp and Schroder, 2010; Guarda et al., 2011) whereas activation of the inflammasome can be orchestrated by IFN-inducible GTP-ases. For example, activation of AIM2 inflammasome was shown to be mediated by guanylate-binding proteins after bacterial infection (Man et al., 2015; Meunier et al., 2015). Several of these genes were shown to be up-regulated in our data sets and have been reported to be overexpressed after experimental infections with other RABV strains (Wang et al., 2005; Li et al., 2016). Using historical samples we have shown that key pyroptotic molecules are differentially expressed after infection with both wild type and attenuated RABV strains, in different experimental models of infection. We hypothesize that pyroptosis is a key mechanism employed by the host to combat lyssavirus infection in the brain with apparently detrimental effect due to a pronounced pro-inflammatory response. The importance of pyroptosis in the pathogenesis of other viral infections has been described. For instance, non-productively infected CD4 T cells die of pyroptosis during HIV infection accounting for the low CD4 T cell counts during AIDS (Doitsh et al., 2014). This pathway could be targeted for novel treatment options in patients with such severe virus infections such as AIDS. Ongoing studies investigate the potential of CASP-1 inhibitors for the treatment of experimental rabies in mice.

Taken together, we have shown that the genomic signature of the brain of DUVV-NL07-infected mice largely overlaps that of brains of mice infected with RABV-PV. We have also provided evidence of several activated pathways during lyssavirus infections that could play a detrimental role in host survival but could also provide leads for innovative treatment options of this invariably lethal disease.

## Ethics Statement

All animal experiments described in this paper have been conducted according to Dutch guidelines for animal experimentation and approved by the Animal Welfare Committee of the Erasmus Medical Centre, Rotterdam, Netherlands (approval number: EMC2061). All efforts were made to minimize animal suffering.

## Author Contributions

PK, BM, and AO designed the experiments. PK, FZ-B, JR, and GA performed the experiments. PK, BM, H-JH, WI, and AA analyzed the data. PK, BM, H-JH, AA, and AO wrote the manuscript.

## Conflict of Interest Statement

The authors have declared that no competing interests exists apart from AO, who is a part-time employee (SCO) of Viroclinics Biosciences B.V. The stated competing interest does not alter the author’s adherence to all the journal’s policies on sharing data and materials.

## Footnotes

1 www.ingenuity.com

## Supplementary Material

**FIGURE S1 |** Key apoptotic markers in DUVV-NL07- and RABV-PV-infected mice. Genomics data were confirmed by qRT-PCR for CASP-3, CASP-9, and TNF-α. Means and standard error of the mean are depicted in the plots. When differences are significant, the *p*-value is given (Mann–Whitney non-parametric test).
