# Supplementary figures and images for: Analysis of Mouse Brain Transcriptome After Experimental Duvenhage Virus Infection Shows Activation of Innate Immune Response and Pyroptotic Cell Death Pathway
Source: Front Microbiol. 2018 Mar 20;9:397. doi: 10.3389/fmicb.2018.00397 (PMC5869263; doi:10.3389/fmicb.2018.00397)

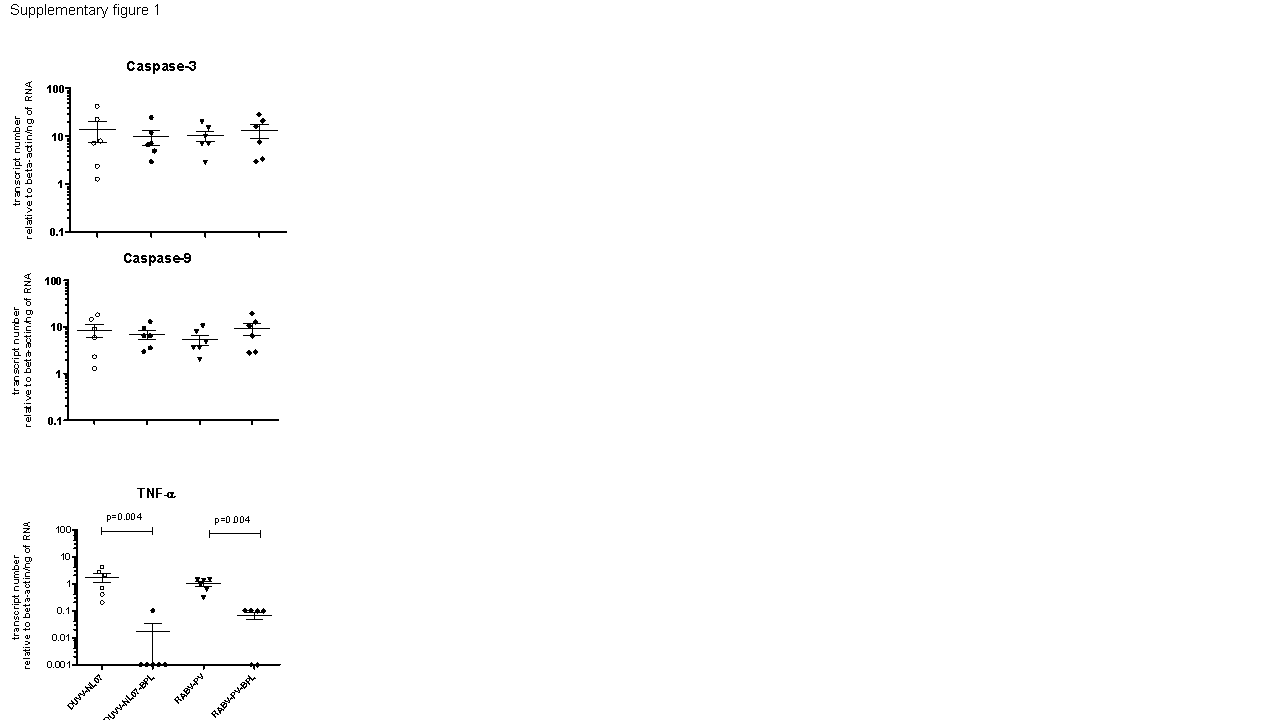

Supplement: Supplementary file 1 [file Image_1.TIF]
